# Diagnosis of Congenital Toxoplasmosis: Challenges and Management Outcomes

**DOI:** 10.7759/cureus.52971

**Published:** 2024-01-26

**Authors:** Ana Losa, Indira Carvalho, Bebiana Sousa, Joanna Ashworth, Ana Guedes, Luísa Carreira, Liliana Pinho, Cristina Godinho

**Affiliations:** 1 Pediatrics Department, Centro Materno-Infantil do Norte, Centro Hospitalar Universitário de Santo António, Porto, PRT; 2 Neonatology Department, Centro Materno-Infantil do Norte, Centro Hospitalar Universitário de Santo António, Porto, PRT

**Keywords:** pediatrics, neonatology, congenital infections, toxoplasma gondii, congenital toxoplasmosis

## Abstract

Introduction

Congenital toxoplasmosis (CT), despite being mostly subclinical at birth, can cause disabling disease in the fetus and lead to long-term sequelae. It is an important cause of chorioretinitis in infants and adolescents. Data on postnatal treatment are controversial, and there is a lack of universal guidelines.

Methods

A cross-sectional study of newborns with suspected CT was conducted between January 2007 and December 2021.

Results

Seventy-one patients with suspected CT were included. During pregnancy, 64 (90.1%) of the mothers underwent therapy, of which 59 (83.1%) with spiramycin. Amniocentesis identified one positive polymerase chain reaction assay. Most newborns were asymptomatic with normal laboratory, ophthalmological, and hearing screening. There was one case of hyperproteinorrachia. Fifty-seven patients (80.3%) started treatment: 42 (73.7%) with spiramycin, seven (12.3%) with pyrimethamine, sulfadiazine, and folinic acid (P+S+FA), and eight (14%) with P+S+FA intercalated with spiramycin. Adverse effects were found in 11 (19.3%) cases, mainly neutropenia. After investigation, we found three confirmed CT cases corresponding to 4.2% of suspected cases and an incidence of 0.4 per 10,000 births. All had normal clinical and laboratory exams in the neonatal period and started P+S+FA, fulfilling 12 months of therapy. During the follow-up, all presented normal psychomotor development without any long-term sequelae.

Conclusion

The lower incidence in our study, compared to the incidence in Europe, may be related to the decline in the prevalence of toxoplasmosis as well as the effectiveness of measures to prevent primary infection and a well-established program of antenatal screening, followed by the early initiation of treatment during pregnancy to prevent vertical transmission.

## Introduction

Toxoplasmosis is caused by infection with *Toxoplasma gondii*, an intracellular protozoan parasite with a worldwide distribution and one of the most common parasitic infections in humans. *T. gondii* infection is most typically asymptomatic, but primary infection in pregnant women can lead to congenital toxoplasmosis (CT). CT results from the hematogenous transmission of the parasite across the placenta and can cause severe and disabling disease in the developing fetus and newborn [1-3].

The risk of fetal infection varies depending on whether the mother receives treatment in pregnancy and increases with gestational age. On the contrary, the risk of severe CT is inversely proportional to gestational age [2,4,5]. Therefore, fetal infections that develop in the early stages of pregnancy may result in negative outcomes like spontaneous abortion or brain damage, whereas fetal infections that occur in the late stages of pregnancy are typically subclinical [6-8].

Although it is subclinical in roughly 75-90% of infected newborns, CT has a wide range of unspecific clinical manifestations. Only a small percentage presents with the complete classic triad (hydrocephalus, intracranial calcifications, and chorioretinitis) [2,3,9]. The most characteristic ophthalmological lesion is focal chorioretinitis, and ocular lesions can develop or recur during childhood and adolescence or even later in life [3,10].

Data on the efficacy of prenatal and postnatal treatment are scarce, and there are no universal guidelines for postnatal treatment. Furthermore, the burden of the disease varies in different countries, and as a result, practices vary between centers [3,11]. There is also no clear consensus on the management of asymptomatic infants in whom CT cannot be confirmed or ruled out during the initial evaluation [11]. In the past, our protocol for treating asymptomatic doubtful cases of CT included treatment with cycles of pyrimethamine, sulfadiazine, and folinic acid (P+S+FA) for four weeks alternating with spiramycin for six weeks, which is no longer recommended because spiramycin is a parasitostatic drug and doesn't have benefits after the infection has occurred. Current treatment regimens include a combination of P+S+FA for one year and seem to be associated with significantly fewer and less severe sequelae [12,13].

This study aimed to characterize the suspected cases and determine the incidence of CT in a tertiary hospital. This research was previously presented as an abstract and e-poster at the 41st Annual Meeting of the European Society for Paediatric Infectious Diseases, held from May 8 to 12, 2023.

## Materials and methods

Study design, settings, and patients

A cross-sectional study was performed with a sample of newborns with suspected CT followed in the Neonatology Department of a tertiary center (Centro Materno-Infantil do Norte, Centro Hospitalar Universitário de Santo António, Porto, Portugal) between January 2007 and December 2021. Inclusion criteria comprised neonates born in our hospital or referred from other hospitals with suspected CT: maternal serology for *T. gondii* with proven seroconversion during pregnancy; positive IgM and IgG with any degree of avidity or positive IgM with negative IgG; a positive polymerase chain reaction (PCR) assay for *T. gondii* in the amniotic fluid; and characteristic symptoms of CT. In contrast, exclusion criteria were cases of suspected CT where suspicion was not supported after an accurate investigation.

Data on the prenatal background (maternal gravidity and parity, maternal toxoplasmosis serology during pregnancy and trimester of seroconversion, amniocentesis and its PCR result, prenatal ultrasounds, and maternal therapy), birth data (type of delivery, sex, gestational age, and birth weight), postnatal period data (symptomatology and physical examination, including ophthalmological exam and hearing screening), postnatal investigation of CT (complete blood count, liver enzymes, cerebrospinal fluid (CSF) analysis, and transfontanellar ultrasound), treatment and its complications data, and follow-up were gathered from the electronic records.

A part of the investigation was carried out in a reference laboratory, involving the direct detection of the parasite through the inoculation of a newborn blood sample and placenta into mice. The results were observed at 10 days, three weeks, and six weeks after inoculation. Additionally, the investigation included a comparative analysis of mother/newborn IgG and IgM using Western blot, as well as a PCR assay conducted on newborn blood.

Ethical approval

This research complies with all the relevant national regulations and institutional policies and is in accordance with the tenets of the Helsinki Declaration. The study was approved by the Clinical Research Department and Ethical Committee of Centro Hospitalar Universitário de Santo António and Institute of Biomedical Sciences Abel Salazar (approval number: 2023.167). In line with recent normative, the requirement to obtain informed consent was waived for this study.

Statistical analysis

Data were collected, processed, and analyzed with IBM SPSS Statistics for Windows, Version 28.0 (Released 2021; IBM Corp., Armonk, New York, United States). Categorical variables were described using absolute and relative frequencies.

## Results

During the study period, there were 49,025 births, and 80 (0.16%) of those had a suspicion of maternal toxoplasmosis during pregnancy. Fourteen cases were excluded, because suspicion was not supported (false-positive cases, unconfirmed maternal seroconversion, and maternal infection prior to conception). Five newborns were referred from other hospitals, resulting in a final sample of 71 suspected CT.

The sample demographics and clinical characteristics of suspected CT cases are shown in Table 1. Regarding maternal gestational history, 33 (46.5%) were primigravida and 38 (53.5%) were primiparous, and prenatal care was performed late or irregularly in four cases. Maternal seroconversion occurred in the first trimester in 39 (54.9%), in the second trimester in 16 (22.5%), and in the third trimester in 12 (16.9%), and it was unknown in the other four cases (5.7%). Maternal therapy until delivery included spiramycin in 59 cases (83.1%) and P+S+FA in five (7%); seven women (9.9%) did not receive treatment. Prenatal ultrasounds were normal in 66 cases (92.9%), with fetal growth restriction (n=1), polyhydramnios (n=2), and unilateral ventriculomegaly (n=2) being the only observed alterations. Thirty-seven (52.1%) mothers were submitted to amniocentesis, and PCR in the amniotic fluid was positive in one (2.7%), negative in 33 (89.2%), and unknown in three cases (8.1%).

**Table 1:** Demographic and clinical characteristics of suspected CT cases. CSF: cerebrospinal fluid; P+S+FA: pyrimethamine, sulfadiazine, and folinic acid; PCR: polymerase chain reaction

Analyzed variable		Suspected CT cases (n=71)
Maternal seroconversion		
First trimester	39 (54.9%)	
Second trimester	16 (22.5%)	
Third trimester	12 (16.9%)	
Unknown	4 (5.7%)	
Maternal therapy	63 (99.1%)	
Spiramycin	59 (83.1%)	
P+S+FA	5 (7%)	
None	7 (9.9%)	
Prenatal ultrasound		
Normal	66 (92.9%)	
Altered	5 (7.1%)	
Amniocentesis	37 (52.1%)	
Amniotic fluid PCR		
Negative	33 (89.1%)	
Positive	1 (2.7%)	
Unknown	3 (8.1%)	
Type of delivery		
Vaginal delivery	45 (63.4%)	
Cesarean	26 (36.6%)	
Sex		
Male	39 (54.9%)	
Female	32 (45.1%)	
Term pregnancy		
Full-term	68 (95.8%)	
Preterm	3 (4.2%)	
Symptomatology		
None	58 (81.7%)	
Jaundice	13 (18.3%)	
Normal audiological examination	70 (98.6%)	
Normal ophthalmologic examination (n=64)	62 (96.9%)	
Started therapeutics	57 (80.3%)	
Spiramycin	42 (73.7%)	
P+S+FA	7 (12.3%)	
P+S+FA intercalated with spiramycin	8 (14%)	
Therapeutics adverse effects	11 (19.3%)	
Neutropenia	10 (17.5%)	
Oral intolerance	1 (1.8%)	

Forty-five (63.4%) neonates were born by vaginal delivery. There were 39 (54.9%) males, three (4.2%) were late preterm, five (7%) were large for gestational age, and two (2.8%) were small for gestational age.

Most of the newborns (81.7%) were asymptomatic in the first days of life. Of the symptoms that can be linked to CT, jaundice was the only manifestation, and it was present in 13 cases (18.3%), six of which required phototherapy. One case of anemia and six cases of significant increase in liver enzymes were found on laboratory work-up that included a complete blood count and liver enzymes in 68 and 66 cases, respectively. CSF analysis was performed in 51 (71.8%) neonates and identified one case of hyperproteinorrachia.

Transfontanellar ultrasound was used for neuroimaging in 68 (95.8%) newborns, showing unspecific and non-typical findings for CT in nine cases. An ophthalmological exam was conducted in 64 (90.1%) and was altered in two cases, but these findings weren't related to CT. The hearing screening was performed in all neonates, being altered in one case.

The inoculation of a mouse with a placenta sample led to the detection of the parasite in two cases (one at 10 days and the other at six weeks after inoculation); the Western blot comparative analysis of mother/newborn IgG and IgM was performed in 37 (52.1%) cases, and it was positive in one; the PCR assay in newborn blood was done in 55 (77.5%) cases, and it was negative in all of them.

Three cases of positive serology for *T. gondii* were found at 12-month follow-up, all of them with positive IgG and one with positive IgM. The time for IgG to become negative was less than two months (n=5), between two and six months (n=25), between six and nine months (n=25), and between nine and 12 months (n=11), and two were lost to follow-up.

Fifty-seven patients (80.3%) started treatment for suspected CT: 42 (73.7%) with spiramycin, seven (12.3%) with P+S+FA, and eight (14%) with intercalated cycles of spiramycin and P+S+FA. The newborn with hyperproteinorrachia was also treated with corticosteroids. Neutropenia was the most frequent adverse effect and occurred in 10 cases (17.5%), all of which were under therapy with P+S+FA. The dose of FA was increased in all these patients, up to 20 mg three times per week, but due to sustained neutropenia, the treatment had to be suspended in one patient. Spiramycin was discontinued in one patient because of oral intolerance. Two negative serologies were obtained prior to treatment discontinuation, and the timing of suspension was less than one month (n=4), after one month (n=23), after two months (n=13), after three months (n=5), after four months (n=1), after five months (n=3), after seven months (n=1), after eight months (n=1), and after 12 months (n=3). Three patients were lost to follow-up.

In conclusion, there were three confirmed cases of CT (one of them was outborn), which corresponds to 4.2% of suspected cases and an incidence of 0.4 per 10,000 births with a vertical transmission rate of 3% in our unit. Table 2 summarizes the clinical presentation, investigation, and management of the three confirmed CT cases.

**Table 2:** Summarizing clinical presentation, investigation, and management of confirmed CT cases. CSF: cerebrospinal fluid; NP: not performed; P+S+FA: pyrimethamine, sulfadiazine, and folinic acid; PCR: polymerase chain reaction

Analyzed variable	Case number		
	Case 1 (2007)	Case 2 (2010)	Case 3 (2015)
Trimester of seroconversion	Third	Third	Second
Maternal therapy	Spiramycin	Spiramycin	Spiramycin
Amniotic fluid PCR	NP	NP	Positive
Clinical examination	Normal	Normal	Normal
Audiological examination	Normal	Normal	Normal
Ophthalmologic examination	Normal	Normal	Normal
Transfontanellar ultrasound	Normal	Normal	Normal
Blood count and liver enzymes	Normal	Normal	Normal
Western blot	Similar	Different	NP
Mouse inoculation with placenta	Positive (six weeks)	Positive (10 days, three weeks, and six weeks)	NP
PCR assay of newborn blood sample	Negative	Negative	NP
CSF cytochemical analysis	Normal	Normal	Hyperproteinorrachia
Serological screening at birth:			
IgG	Positive	Positive	Positive
IgM	Negative	Negative	Negative
Therapeutics	P+S+FA and spiramycin	P+S+FA, altered to spiramycin	P+S+FA and corticotherapy
Therapeutics adverse effects	Neutropenia	Neutropenia	None
First months of serological screening evolution	Progressive decrease of IgG	Rising IgG levels after six months	Sustained positive IgG
Serological screening evolution after therapeutic suspension:			
IgG	Positive	Positive	Positive (rising levels)
IgM	Positive	Negative	Negative
Treatment duration	12 months	12 months (irregular compliance)	12 months
Clinical and psychomotor development	Asymptomatic	Asymptomatic	Asymptomatic
Follow-up	12 months	Seven years	Four years

## Discussion

Epidemiology and transmission

There is a wide disparity between countries in the incidence of CT, which is estimated to range from 0.1 to six cases per 1000 live births. The highest burden of CT is in the Middle East and South America, and the incidence in Europe is estimated to range from 0.5 to 1.6 per 10,000 and in France from two to four per 10,000 live births [3,12,14]. In our study, we found three confirmed cases of CT, which corresponds to 4.2% of suspected cases, and an incidence of 0.4 per 10,000 births, which is slightly below the range estimated for Europe and lower than the incidence of 5.6 per 10,000 live births reported in a previous Portuguese study [15]. Some genotypes are more virulent and may contribute to justifying these geographical differences throughout the world, and in addition, some are also more correlated with chorioretinitis.

*T. gondii* can be transmitted to humans by consumption of raw and poorly cooked meat containing viable tissue cysts, ingestion of water or food contaminated by oocysts excreted in cat feces, or vertical transmission during pregnancy [12,16]. The seroprevalence of *T. gondii* has been declining, particularly in developed countries, and this trend can be attributed to a number of factors, including increased frozen meat consumption, modern mass-produced meat production, improved hygiene, urbanization, etc. [9]. In Portugal, according to the latest epidemiological data, the seroprevalence of *T. gondii* infection has been declining, from a rate of 47% in 1979 to 18% between 2009 and 2020. Therefore, approximately 80% of women of childbearing age are susceptible to toxoplasmosis infection during pregnancy, with a risk of CT in the newborn [17]. It is essential to know the proportion of susceptible pregnant women in any given area to establish the best strategy for the local prevention of CT.

Prenatal surveillance

In the literature, it is described that the risk of vertical transmission increases directly proportional to gestational age at the time of primary maternal infection and may be reduced by antenatal treatment [12]. In our research, most (54.9%) of maternal seroconversion occurred in the first trimester, which is associated with a lower probability of placental transmission and can contribute to the lower number of cases of CT, with a vertical transmission rate of 3%. Spiramycin was the most used drug, and it was also the drug used by all three mothers of newborns with confirmed CT. However, currently, spiramycin is used for preventing fetal infection, and P+S+FA is reserved for the treatment of confirmed fetal infection or when fetal infection occurs in the third trimester and amniocentesis is not performed, due to the high fetal risk.

Postnatal period

The majority of CT cases, according to the literature, are asymptomatic, and in our study, the three confirmed cases were asymptomatic.

In CT, CSF analysis can reveal hyperproteinorrachia or mononuclear pleocytosis, and the detection of *T. gondii* by PCR can confirm the diagnosis. We found one case of hyperproteinorrachia, which corresponded to a CT-confirmed case, but PCR of CSF is not usually performed in our center.

In Europe, neuroimaging with transfontanellar ultrasound is more frequently used, which relates to the widespread availability and absence of radiation, despite computed tomography being described as having a higher sensitivity for the detection of calcifications [3]. In our center, the neuroimaging exam used in all cases was transfontanellar ultrasound, and no calcifications or other typical findings of CT were found.

Specific investigation

A CT diagnosis can be reached through the combination of the following: newborn positive IgG and IgM for *T. gondii*; newborn IgG with a significantly higher value than maternal IgG at birth; positive PCR assay in the amniotic fluid; positive PCR assay in newborn blood or CSF; or the persistence of positive IgG after 12 months, which is the gold standard. However, interpretation of serologic testing can be challenging for the following reasons: positive newborn IgG can reflect past or current infection in the mother since it crosses the placenta; small amounts of maternal IgM can cross the placenta, resulting in low IgM levels in uninfected newborns if performed soon after birth; otherwise, if infection happens late in pregnancy, cases of false-negative IgM may occur, as IgM appears within 1-2 weeks of exposure; and maternal therapy can alter the serological profile of the newborn since it can delay IgG production [12].

The support of a reference laboratory is fundamental for an accurate diagnosis of CT, and in our three confirmed cases, all had positive findings in the investigation carried out in this laboratory: a positive result after inoculation in the mouse of a blood sample or placenta (n=2), a different pattern in Western blot (n=1), and detection of *T. gondii* in the amniotic fluid with a PCR assay (n=1).

After discontinuation of treatment, a rebound rise of IgM and/or IgG for *T. gondii* may be seen in CT cases, and this situation is thought to be due to a delayed serological response to infection rather than a relapse. Therefore, a positive IgG beyond 12 months of age is considered the gold standard and confirms a diagnosis of CT [12]. In our study, the three cases of CT had positive IgG at 12 months, and one had concomitantly positive IgM.

Treatment and adverse effects

It is fundamental to weigh the risks and benefits before starting therapy in suspected or inconclusive cases since therapy can be associated with adverse effects. There is no clear consensus on treating asymptomatic cases, and in some studies, clinicians consider monitoring serology while postponing treatment [12,18].

Complete blood counts and liver enzyme tests must be performed prior to initiating treatment, and subsequently, adverse effects must be monitored. Hematological toxicity is the principal adverse effect of pyrimethamine and sulfadiazine, and neutropenia is described as occurring in more than half the children treated with these drugs, but the addition of folinic acid reduces the likelihood of this adverse effect [3]. In our study, of the 15 patients who did P+S+FA, neutropenia occurred in 10 (66.7%), and all but one resolved after increasing the dose of FA. Two cases of confirmed CT developed neutropenia after treatment with P+S+FA, and none had hepatotoxicity. 

Follow-up

In our study, none of the three cases developed chorioretinitis during follow-up. However, patients with CT can be asymptomatic until the second or third decade of life, when lesions develop in the eye, presumably due to cyst rupture and the subsequent release of tachyzoites and bradyzoites [2]. Nevertheless, regular follow-up in an ophthalmology consultation is recommended to be maintained due to the possibility of the occurrence or recurrence of chorioretinitis at least to an age when the child can identify significant visual changes and, according to some studies, annually until the second decade of life [2,10,19,20].

Strengths and limitations

The major strength of this study was the 15 years of data collection, which allowed for a comprehensive characterization of newborns with suspected CT at a tertiary pediatric hospital. However, this study has some limitations. Firstly, like any retrospective study, it has inherent limitations associated with data collection from medical records, including missing or incomplete information. Secondly, the low number of confirmed CT cases and the absence of a control group limit the ability to make direct comparisons or draw definitive conclusions regarding the positive cases. Despite these limitations, the study provides valuable insights into the clinical and investigation aspects as well as the therapeutics and complications associated with suspected CT.

## Conclusions

The low incidence of CT in our study may be related to the decline in the prevalence of toxoplasmosis observed in our country, as well as the effectiveness of measures to prevent primary infection and a well-established program of neonatal screening, followed by the early initiation of treatment during pregnancy to prevent vertical transmission. New guidelines must be developed to avoid the high number of cases of unconfirmed CT that are submitted to therapy, whose adverse effects cannot be neglected, as documented in our study. Furthermore, the available evidence on CT is limited and relies essentially on observational studies. In the future, new studies are needed to determine the global impact of congenital toxoplasmosis, as well as the efficacy of postnatal treatment in preventing the emergence of late sequelae in asymptomatic cases.
